# Characterization of strictly lytic phages infecting *Oenococcus oeni* from Merlot wines and proposal of a new genus

**DOI:** 10.1128/spectrum.02588-24

**Published:** 2025-08-12

**Authors:** Yasma Barchi, Florencia Oviedo-Hernandez, Amel Chaïb, Cécile Philippe, Olivier Claisse, Christian Cambillau, Adeline Goulet, Claire Le Marrec

**Affiliations:** 1 Université Bordeaux, INRAE, Bordeaux INP, Bordeaux Sciences Agro, UMR1366 Œnologie, F-33140, Villenave d'Ornon, France; 2 School of Microbiology & APC Microbiome Ireland, University College Cork 8795, Cork, Ireland; 3 Laboratoire d'Ingénierie des Systèmes Macromoléculaires (LISM, UMR7255), Institut de Microbiologie de la Méditerranée, Aix Marseille Université, Centre National de la Recherche Scientifique, Marseille, France; Université de Lorraine, Nancy, France

**Keywords:** lytic phage, *Oenococcus*, winemaking, malolactic fermentation, AlphaFold2, host adhesion device, domain shuffling

## Abstract

*Oenococcus oeni* is the predominant lactic acid bacteria species in wine, where it performs the malolactic fermentation, which helps to secure and preserve wine quality. Here, we describe the morphological, biological, and genomic characterization of siphophage Krappator X_27_, a strictly lytic phage that was previously isolated from Merlot wines. Several aspects of the life cycle of the phage were investigated using the sensitive strain IOEBS277 under optimal growth conditions. X_27_ has a large burst size (149 Plaque Forming unit [PFU] per infected cell) and targets industrially relevant strains of *O. oeni* used for production of wines. X_27_ also shows lytic activity against its host in red and white grape juice media. The phage genome consists of 41,633 nucleotides. Structure prediction of the viral adhesion devices reveals elongated multi-domain machinery containing carbohydrate-binding modules likely involved in host recognition and binding. Genome-based phylogeny revealed that X_27_ has less than 30% intergenomic similarities with its most similar phages and represents a new species in a yet unassigned genus, for which we propose the name « Krappavirus *»*. Aiming to expand the available collection of genomes in this genus, we sequenced two independently isolated phage homologs from Merlot wines. Modular protein evolution may play a role in the diversification of this specific phage lineage, and naturally occurring domain combinations were found in three crucial proteins of known function: the small terminase subunit, the endolysin, and the replisome organizer.

**IMPORTANCE:**

*Oenococcus oeni* is commonly used for wine and cider production. Characterizing strictly lytic oenophages, understanding their genetic relationships, and studying their interactions with various hosts are the necessary steps for preventing and controlling phage attacks that occur along the fermentation process.

## INTRODUCTION

Wines are among the most iconic fermented products worldwide. The conversion of grape juice to wine relies on yeasts and lactic acid bacteria (LAB) that conduct the alcoholic fermentation (AF) and the malolactic fermentation (MLF), respectively (1, 2). The ecology of wine fermentations at the scale of wineries has revealed a large diversity of microorganisms including various yeasts, bacteria, and fungi. Further exploration has then expanded to the whole vineyard, which represents the initial environment affecting the microbial makeup found in wine fermentation. Acquisition of such data is essential to preserve and improve wine quality, anticipate climate change, and assist winemakers during the transition toward more sustainable practice, such as the reduction of sulfites and implementation of biocontrol strategies.

In recent years, multi-omic tools have become increasingly important to better understand the function and resilience of vine-wine ecosystems. High-throughput analyses have recently questioned how geography, climate, soil properties, and winemaking practices may impact and shape microbial communities (3). A comprehensive understanding of the vine-wine microbial ecosystem also requires the consideration of the virosphere. Viruses infecting bacteria, namely bacteriophages or phages, have so far drawn a great deal of attention during the winemaking steps. Phages are widespread in must (crushed grapes) and wines where they infect spoiling bacteria, such as acetic acid bacteria (4), as well as the beneficial LAB *Oenococcus oeni* that drives MLF (5). An increasing amount of data on the diversity and abundance of *O. oeni*-infecting phages (oenophages) is becoming available. Oenococcal strains of commercial or indigenous origin are described as typically harboring a number of active prophages in their chromosomes (6, 7). The prevalence of lysogeny that was observed during *in silico* exploration of sequenced genomes was further corroborated during *in vivo* studies of the dynamics of the population upon completion of spontaneous MLF. Prophage carriage in *O. oeni* may therefore impart both positive and negative attributes in this specific industrial context (5).

The viral population associated with winemaking also consists of ex-temperate phages such as OE33PA, and strictly lytic phages of the Vinitor lineage (6, 8, 9). The latter phages are particularly abundant in musts and may therefore participate in the selection of strains responsible for MLF during the early steps of winemaking.

Comparative genomics of over 250 fully sequenced viral genomes shows that oenophages can be divided into two groups of closely related genomes referred to as clusters, which can be further divided into subclusters (6). The cluster I phages are exclusively temperate, or ex-temperate, while cluster II contains both temperate (Int_D_ group, recently assigned to the genus *Sozzivirus* by International Committee on Taxonomy of Viruses [ICTV]) and strictly lytic (Vinitor) oenophages. Interestingly, Vinitor phages exhibit a unique evolutionary trajectory, and their gene repertoire contains sequences related to epiphytic and/or insect-associated LAB (8–10).

The observed modular and combinatorial nature of oenophage genomes was exploited to develop a PCR typing system for rapid assessment of diversity among oeno-phages (6, 8, 9, 11). It was used to screen the novelty and diversity of a collection of previous phage isolates, stored year after year (11). We observed that the genome of Krappator X_27_ (hereafter “X_27_”) did not amplify the conserved regions along currently described oenophages, suggesting that X_27_ may represent another lineage of oenophages (11). Here, we report the morphological, biological, and genomic characterization of this strictly lytic oenophage isolated from a Merlot wine. Using a PCR specific for the *tmp* gene of X_27_, we identified two additional phage homologs in wines made from the same grape variety. The three described Krappator phages represent two species in a yet unassigned genus, for which we propose the name « Krappavirus ». Altogether, genomic comparisons also suggest that the isolated oenophages are able to shuffle fragments of three key proteins, which may facilitate adaptation to their hosts.

## RESULTS

### Physiological characteristics and morphology of Krappator X_27_

Phage X_27_ was isolated from a Merlot red wine collected in 2015 in France (8). It was easy to propagate, and infections at multiplicity of infection (MOI) >10^−6^ caused complete lysis of strain IOEBS277 in MRS_Φ_ (MRS agar supplemented with MgSO4 (3.75 g/l) and CaCl2 (2.375 g/l)) broth at 25°C, following 72 h incubation. The phage produced maximum titer (2 × 10^10^ PFU/mL) for MOIs ranging from 2.5 × 10^−4^ to 10^−3^ (Fig. S1). Lysates could be stored at 4°C for extended periods of time with a 2-log reduction in phage titer (8.75 × 10^9^ to 6.25 × 10^7^ PFU/mL) within 16 months.

**Fig 1:**
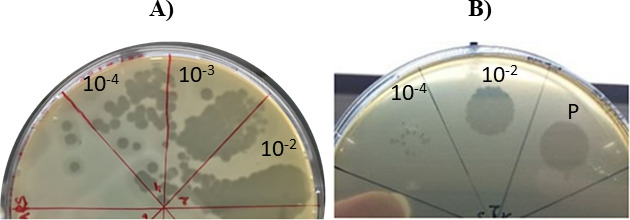
Titrations of lysates of the X_27_ (**A**) and Vinitor 162 (**B**) phages on *O. oeni* IOEBS277. Image B is adapted with permission from reference (9). Volumes of 8 µL of the pure (**P**) and serial dilutions were spotted on the lawns of the host strain.

X_27_ produced large and clear plaques on the lawns of its host (Fig. 1A), which were easily distinguishable from those produced by the strictly lytic Vinitor oenophages (Fig. 1B). A distinctive turbid halo zone around plaques was observed for X_27_, whose width incrementally increased over time. This has previously been described as an indicator of phage-associated exopolysaccharide depolymerization (12).

Host adsorption and one-step growth curve tests were performed in MRS_Φ_ broth. An 85% host adsorption efficiency was reached in 60 min at 25°C. The latent period was 3 h, and newly synthesized particles were released after an additional burst period of 2 h. The burst size was 149 ±  4 PFU/infected cell (Fig. S2).

**Fig 2:**
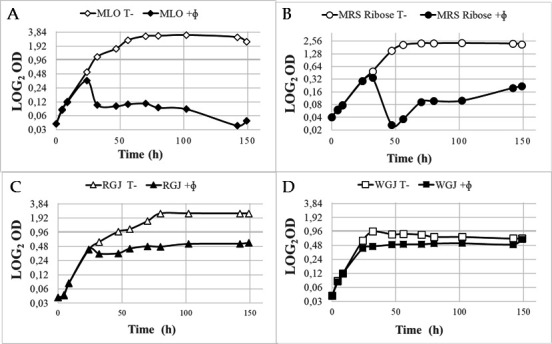
Impact of medium composition on the infection of strain IOEBS277 by X_27_ at an MOI of 0.003. Growth curves of the control and infected cultures in MLO (**A**), MRS Ribose (**B**), Red Grape Juice (RGJ) (**C**) and White Grape Juice (WGJ) broth (**D**). Time zero represents the time of phage addition.

Phage infection was also efficient in other liquid media supporting the growth of *O. oeni*, such as Medium for Leuconostoc oenos (MLO) and modified MRS with ribose replacing dextrose (Fig. 2A and B). Ribose is one of the major residual sugars in wine after completion of AF. In contrast, infection was less efficient in Red Grape Juice liquid medium and was barely observed in White Grape Juice broth (Fig. 2C and D).

A host range test was carried out for a panel of 20 *O. oeni* strains representing the four main phylogenetic groups described in the species (Table 1). Half of the tested bacterial strains were susceptible to X_27_. These sensitive strains belonged to three phylogroups (A, C, D) and were associated with wine, cider, and kombucha. Efficiencies of plaquing (EOP) and/or plaque sizes were reduced on six sensitive strains, suggesting the presence of active phage resistance mechanisms. Previous *in silico* studies showed that genes coding for restriction-modification and Sie systems are widespread in *O. oeni,* while the species completely lacks CRISPR-Cas systems (6, 11). Conversely, X_27_ did not form any plaques on 10 strains, including all four tested strains from phylogroup B. In this small set, LAD2 was remarkable as its infection by X_27_ at high MOI produced a clear lysis zone in the first two dilution spots, with no individual plaques, nor any sign of lytic activity in further dilution spots. The lysis zone still occurred when spotting the same PFU of a dialyzed lysate and was not dependent on phage lysate-contaminating lysins or bacteriocins. Bacterial killing may have therefore occurred by phage binding that does not lead to a productive infection. The exposure of LAD2 to high-multiplicity virion adsorption with lytic action of phage structural proteins may degrade the peptidoglycan at multiple sites, leading to “lysis from without” (12, 13). Alternately, LAD2 may have another specific abortive mechanism as compared to the other resistant strains.

**TABLE 1:** X_27_ phage production on different *O. oeni* strains as represented by EOP values

Tested strains and origin		Phage production^*a*^ on relevant strains^*b*^		
		High 10^−1^ < eop < 1	Medium 10^−7^ < eop < 10^−1^	None eop < 10^−8^
*O. oeni*	Wine	PSU-1, IOEB1491, LAA4 (A)	IOEB0608, LAB6, IOEBS25, LAB2013 (A)	IOEBCiNe, S28, VF, LAD2, CI4 (A); IOEBB10, IOEB9805, IOEB8413 (B)
	Cider	CRBO1381 (C)	C52 (C)	C23 (B) ; CRBO1384 (C)
	Kombucha		BL4 (D)	
*Oenococcus sicerae*	Cider			UCMA15228
*Oenococcus kitaharae*	Shochu			NRIC0647
	Water kefir			CRBO2176
*Oenococcus alcoholitolerans*	Cachaça			JP736 ; JP72/2

^*a*^ High EOP values represent sensitive strains and medium EOP values suggest the presence of bacterial resistance mechanisms. EOP values less than 10^−8^ indicate that the tested strains are fully resistant to X_27_.

^*b*^ Phylogroup membership (A to D) in the *O. oeni* species is given in brackets.

Testing strains of the *O. sicerae*, *O. kitaharae,* and *O. alcoholitolerans* species showed no plaques, suggesting that X_27_ was not capable of cross*-*taxonomic order infectivity (Table 1).

### Genome characterization of X_27_

**Fig 3:**
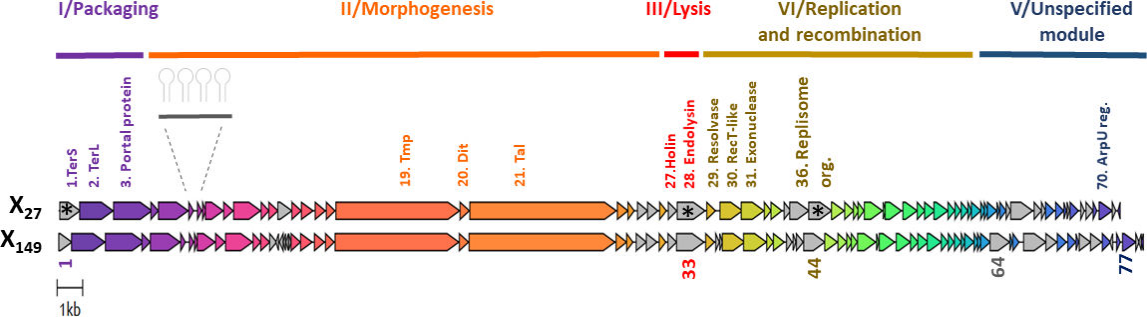
The genome comparison of X_27_ and X_149_ was performed and visualized with Clinker (14). The first base of small terminase subunit (terS) genes was selected as the starting point of the genomes. The functional gene groups (modules I to V) are indicated in different colors. Genes in gray have an identity level below 90%. The three ORFs encoding shuffled proteins are indicated with an asterisk.

The X_27_ genome consists of 41,633 bp with a GC content of 36.8%. It carries 72 putative open reading frames (ORFs), which are all positioned on the same DNA strand, except ORF72 (Fig. 3). All genes, except for ORF1, use an ATG start codon. The identified ORFs code for proteins that range between 30 and 1,619 amino acids (aa) in size. Putative functions were assigned to 30 ORFs (41.7%) using BLASTp National Center for Biotechnology Information (NCBI), Prokaryotic Virus Remote Homologous Groups (PHROG), and HHpred (Table S1). No gene coding for an integrase, excisionase, or repressor gene could be identified, confirming that the X_27_ life cycle is strictly lytic.

The genome is organized into five functional modules (I–V) similar to those described for other oenophages known to date (5). Following annotation convention, the genome starts with DNA packaging genes (ORF1–ORF2) encoding the small and large subunits (TerS and TerL) of a terminase complex (Fig. 3). The packaging module also contains the gene coding for the portal protein. TerS_X27_ shares 71%–72% sequence identity with corresponding proteins found in *Sozzivirus* and Int_E_ oenophages (6). An HHpred analysis identified domains similar to the DNA-binding domain of TerS from *pac* packager phages, such as Sf6 infecting *Shigella flexneri* (Prob.: 99.31%, E-value: 6e-11) (15). The TerL_X27_ protein is also most similar to that of well-studied phages with known *pac* DNA packaging strategies, such as PBSX-like phages in *Lactococcus lactis* and *Enterococcus raffinolactis* and the *Lactiplantibacillus plantarum* Silenus phage (16) (Fig. S3). The X_27_ genome is therefore suggested to have circularly permuted termini. Headful-packaging phage *pac* sites usually lie within or near the small terminase subunit gene, and packaging proceeds in the direction in which that gene is transcribed.

The morphogenesis module II consists of 23 genes. The head morphogenesis genes with the predicted functions include minor capsid protein (X_27__gp5), major head proteins (X_27__gp10 and X_27__gp11), head scaffolding protein (X_27__gp9), putative maturation head protease (X_27__gp4), head-tail adaptor (X_27__gp12), and head-closure-knob protein (X_27__gp13) (Fig. 3). The module also includes three orphan genes (ORF6–8). Yet, the corresponding 650 bp-nucleotide region is also predicted to contain four putative Rho-independent terminators, interspaced by 55–72 bp sequences, resembling small non-coding RNAs produced by lytic phages to regulate various viral processes (17).

A group of 10 genes (ORF12‒21) forms the tail morphogenesis module. Functionally assigned tail genes include putative tail completion proteins (X_27__gp14, X_27__gp15), major tail protein (MTP, X_27__gp16), tail assembly chaperone (X_27__gp17), tape measure protein (TMP, X_27__gp19), distal tail protein (Dit, X_27__gp20), and tail-associated lysozyme (Tal, X_27__gp21) (Fig. 3). Of note, the TMP comprises a small domain (122 aa) similar to a transglycosylase-like domain found in phage tail fibers (Cd13402; Prob.: 99.16%, E-value: 6.2e − 10) that catalyzes cleavage of the β-1,4-glycosidic bond between the peptidoglycan N-acetylmuramic acid and N-acetyl-D-glucosamine moieties, as does egg-white lysozyme. All phage morphogenesis proteins were largely aligned with their orthologs from Int_D_ temperate oenophages and prophages of the LAB *Liquorilactobacillus satsumensis,* which are associated with fermented beverages such as shochu, kombuchas, and kefirs (18, 19).

Module III is the lysis module and contains ORF28 coding for an endolysin (Lys_X27_). We found no signal peptide involved in its secretion, unlike the well-known fog44 lysin (20). Lys_X27_ has a modular structure comprising an N-terminal enzymatically active domain (EAD) that belongs to the glycoside hydrolase 25 (GH25) family and is suggested to hydrolyze the β-1,4-glycosidic linkage in bacterial cell walls (Fig. 3). A similar domain is found in orphan lysozyme-related protein sequences in a variety of *O. oeni* and *Weissella* sp. strains, as well as in prophages associated with human metagenomes (55%–57% identity) (21, 22). The predicted C*-*terminal cell wall-binding domain used for peptidoglycan recognition (22) had 87.8% identity to the endolysins from the Vinitor oenophages. We found fewer homologs for the EAD section in databanks, and bioinformatic analysis was unable to identify a functional domain.

Lysis systems in gram-positive hosts require a holin, which allows the endolysin to access the host cell wall. With few exceptions (23), holin-encoding genes overlap or are adjacent to lysin-encoding genes. Intriguingly, BLAST analyses of genes upstream and downstream of ORF28 did not retrieve any holin-like protein orthologs, and globally, the deduced proteins aligned poorly with existing phage records. Holins are small proteins containing one, two, or three transmembrane domains/helices (16, 21) and a charged C-terminal region. TMHMM search predicted the putative protein of ORF27 (111 amino acids) as the most likely candidate. It would correspond to a type III holin structure with a single transmembrane region, an N-terminal region located in the periplasm, and a C-terminus located in the cytoplasm.

Module IV contains genes associated with the replication of the phage genome, including a putative replication*-*repair nuclease (ORF29), a replisome organizer (ORF36), two HNH endonucleases (ORF35, ORF41), and a single-strand annealing protein (SSAP) of the RecT/Redβ family (ORF30). The latter is related to an SSAP harbored by an Int_F_ prophage associated with a strain collected from kombucha (6) (46% identity, E-value: 8e − 71). The Best BLAST Hit (BBH) outside of *O. oeni* is to a RecT protein of *Lc. lactis* with 44% identity (WP_259749449.1) (Fig. 3). Phage SSAPs can be involved in different aspects of DNA metabolism throughout phage propagation (24, 25) and also substitute the classical bacterial recombinase RecA to allow recombination between dissimilar sequences. Hence, SSAPs are thought to facilitate genetic transfers between distant phages and play an important role in the extensive genome modularity and mosaicism of their genomes (24–27). As observed in *Bacillales* and *Lactobacillales* phages, two specific functions encoded in direct proximity to the phage-encoded *recT/red*β genes include a Cas4-like nuclease with a PD-(D/E)-XK domain (ORF32) and a single-strand binding protein (ORF33) (24).

No clear delineation could be identified between module IV and the rest of the genome. Therefore, we arbitrarily grouped the right-end genes (ORF56-72) together into module V (Fig. 3). Overall, no functions could be assigned to most proteins using either HHpred or PFAM analyses. Only a conserved domain of ArpU-like transcriptional regulators was found in the gp70 protein. The deduced proteins from ORF47 and ORF55 resemble HNH homing endonucleases, which are often selfish genetic elements that exist free-standing or associated with inteins or introns (28). BBH was the putative NUMOD4 domain-containing protein of *Paenibacillus mucilaginosus* (E-value: 5e − 18; 48% identity; locus tag B2K_02705) and the HNH endonuclease of the *L. plantarum* phage Satyr (E-value: 4e − 84; 68% identity; locus tag: HOS71_gp029). We joined the two ORFs framing each HNH homing endonuclease and performed a BLASTn analysis. None of the resulting hits spanned along the joint region, suggesting that no gene was spliced by these HNH endonucleases.

### The X_27_ host adhesion device is a long fiber-like, multi-domain assembly

**Fig 4:**
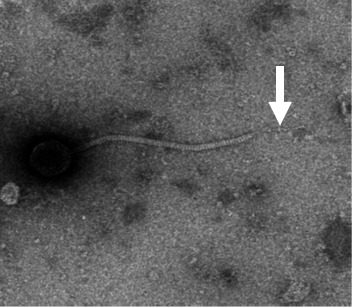
Visualization of phage X_27_ by transmission electron microscopy (TEM). The white arrows show a terminal tail fiber.

Transmission electron microscopy images revealed a preclassification of phage X_27_ into the class of *Caudoviricetes* (29), characterized by an icosahedral capsid of 57.5 ± 0.5 nm in diameter attached to a flexible tail of 246 ± 4 nm in length (Fig. 4). This is the most widespread taxonomic classification among currently characterized LAB phages, bearing in mind that most of them are of dairy origin (30). At the tail distal end, the thin and elongated host adhesion device, involved in the specific binding of yet unidentified receptors in the host cell wall, was overall similar to that of Vinitor 162 (9).

**Fig 5:**
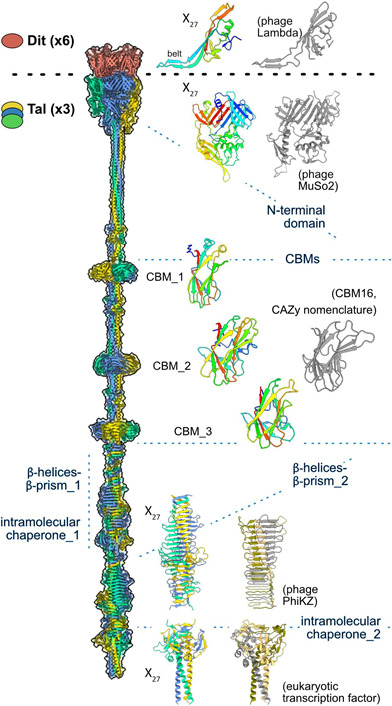
AF2 predicted structures of the X_27_ Dit and Tal proteins. The hexameric ring of Dit is shown as salmon ribbons and transparent surfaces. One Dit monomer is shown as a rainbow ribbon side by side with the crystal structure of the phage Lambda Dit protein (gray ribbon, PDB 8xcg). The trimer of Tal is shown as ribbons and transparent surfaces. A monomer of the Tal N-terminal domain and of every CBM are shown as rainbow ribbons side by side with their closest structural homologs identified by the Dali server (PDB ID 3cdd for the Tal of phage MuSo2, PDB ID 2zew for CBM16). The Tal C-terminal β-helices, β-prism, and intramolecular chaperone are shown as trimeric assemblies. Similar assemblies from the phage PhiKZ (PDB ID 6orj) and the eukaryotic MyRF (PDB ID 7dc3) are shown for comparison.

In order to gain further insights into the host adhesion devices of X_27_, we used AlphaFold2 (AF2), which is well adapted for structural analyses of elongated and flexible assemblies (31–36). AF2 predicted the structure of a hexamer of Dit and a trimer of Tal (Fig. 5). The hexamer represents the common core of siphophage host adhesion devices which interacts on one side with the last ring of MTP proteins and, on the other side, with a trimer of Tal (31). A search for structural homologs in the Protein Data Bank (PDB) using the Dali server revealed that the short Dit (121 aa) of X_27_ is devoid of carbohydrate-binding motif (CBM) and composed of a single domain, like the Dit of the *Escherichia coli* siphophage Lambda (Table S2), referred to as the belt domain, because of its canonical belt loop assembling the hexameric ring (35–40) (Fig. 5; Fig. S4 and S5).

The long Tal of X_27_ (1,914 aa) comprises a bulky N-terminal domain followed by a C-terminal extension (Fig. 5), which is in agreement with the fiber-like structure observed at the tail distal end (Fig. 4). Its N-terminal domain (1–340) is the typical structural domain of Tal proteins, which was first described in the baseplate hub protein gp27 of the myophage T4 (41), assembling host adhesion devices as well as phage tail-related bacterial contractile injection systems (Table S2) (42). Then, the Tal extension consists of a long α-helical rod (aa 421–1,225) containing three CBMs (Fig. 5; Table S2) likely involved in the recognition of *O. oeni* cell wall polysaccharides (CWPs). Lastly, the Tal extension ends with two similar regions (aa 1274–1,553 and 1,565–1,914). Each of them is made up of triple β-helices, β-prisms, and an intramolecular chaperone-like domain (Fig. 5; Table S2). This organization is commonly found in phage tail spike and fiber proteins (43–45). Of note, while tail-associated chaperones possess long β-hairpins contacting the upstream β-prism (44, 45), the X_27_ chaperones, like that of Vinitor 162, are devoid of such tentacles and are structurally closer to the corresponding domain of the eukaryotic MyRF factor (Fig. 5; Fig. S6) (46). These intramolecular chaperones mediate the trimerization and proper folding of their upstream domains and are then auto-proteolytically released to leave mature proteins (45, 46). Serine-lysine catalytic dyads, which are indispensable for their self-cleavage (45, 46), are present in each X_27_ intramolecular chaperone-like domain (Fig. S6). However, the presence of two consecutive intramolecular chaperones in the X_27_ host adhesion device raises questions about their roles in the assembly and function of the Tal.

### X_27_ is the prototype of a new genus provisionally named “Krappavirus”

**Fig 6:**
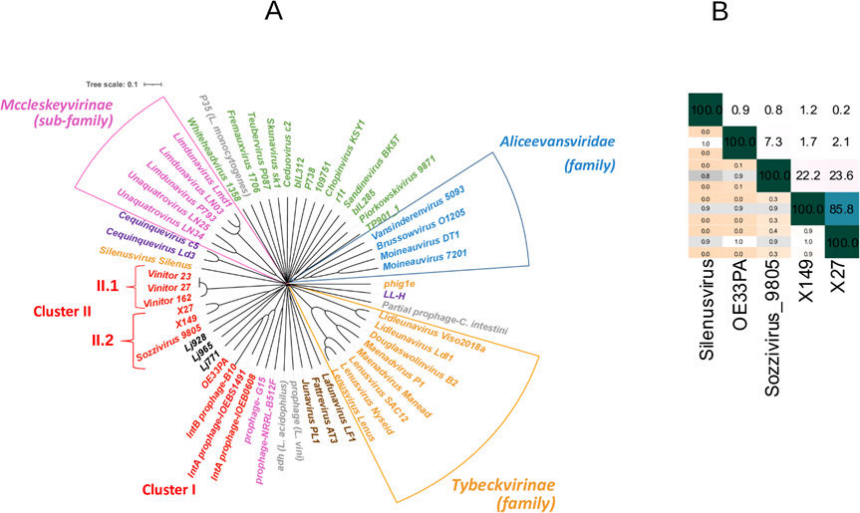
Krappator phages X_27_ and X_149_ correspond to two species of a novel genus. (A) Phylogenetic tree generated by VICTOR using the complete genome sequences of phages infecting LAB genera including X_27_ and X_149_ and eight other phages of *O. oeni* from clusters I and II; (B) heatmap integrating the intergenomic similarity values between oenophages X_27_, X_149_, OE33PA, 9805, and the *L. plantarum* silenusvirus silenus phage, calculated with VIRIDIC. Current nomenclature of phages by ICTV is used. Accession numbers are as reported previously (9).

The genome sequences of X_27_ and related phages (8 known oenophages and 48 other LAB phages) were analyzed using the Virus Classification and Tree Building Online Resource (VICTOR) (47). The analysis positioned X_27_ in *Oenococcus* phage cluster II. Yet, the unclassified branch represented by X_27_ was distant from that of the Vinitor phages, the first group of lytic phages characterized to date (9). Both group*s* exist as two evolutionary lineages which were assigned to subclusters II.2 and II.1, respectively (Fig. 6A). We took a closer look at genome diversity within subcluster II.2 (Fig. 6B). The genome of X_27_ was most closely related to those of temperate phages of the Sozzivirus genus, as well as a prophage from *O. sicerae*. Worth mentioning was the observed conservation of the packaging module among members (Fig. 3). Phages within subcluster II.2 were not restricted to a single genus, and the VICTOR tool suggested that X_27_ represents a novel viral genus by itself. The taxonomic relationships of the X_27_ and Sozzivirus 9805 to each other were also determined by uploading the genomes to Virus Intergenomic Distance Calculator (VIRIDIC) (48). VIRIDIC aligns similar genomes and identifies genus relationships at ≥70% similarity and species at ≥95%. The X_27_ genome displayed 23.6% intergenomic similarity with that of Sozzivirus 9805 (Fig. 6B). Based on the above, we propose that X_27_ defines a representative species of an unassigned genus of siphophages, which we provisionally named “Krappavirus” (Krappa, for grapes in “francique,” an Old Frankish language) (49).

The X_27_ genome sequence was used to design specific primers to examine uncharacterized oenophages collected during previous surveys and stored inside infected cells at −80°C (5, 8). The strategy led to the sequencing of two independently isolated homologs, namely X_28_ and X_149_ phages. Like *X_27_,* they came from Merlot red wines. Altogether, the three phages were obtained from distinct fermentation steps (must, AF, and MLF for X_27_, X_28_, and X_149_, respectively) and during two consecutive millesimes (2014 for X_149_ and 2015 for X_27_ and X_28_). The X_27_ and X_28_ genomes shared 99.9% sequence identity at the nucleotide level, as tested reciprocally, and contained the same number of predicted ORFs. X_27_ and X_28_ therefore corresponded to variants of the same phage species. In contrast, the genome of X_149_ was slightly larger with 42,545 bp and had 80 predicted ORFs. Although both genomes showed near-complete collinearity, some dissimilarities mapped to two genomic segments (Fig. 3). Importantly, both VICTOR and VIRIDIC classified X_27_ and X_149_ as separate species (intergenomic identity 85.8%) (Fig. 6).

The most variable region between both phages was module V, and an apparent gain of certain ORFs was evident in the X_149_ genome. They included ORF64_X149_ coding for an adenine-specific methyltransferase, with identified orthologs in the genomes of various human metagenome-associated phages (22), lactophages (50), as well as uncharacterized prophages of Fructilactobacilli. Automated annotation also identified four additional small ORFs (17–20) in X_149_ in module II that are potentially involved in the connection of the phage head and tail (Fig. 3). Although both Dit proteins were 100% identical, Tal components shared 96% identity (Fig. S5). Amino acid changes are distributed in the whole sequence, especially in the region spanning the potential saccharide-binding pocket of the first CBM (Fig. S5). The host range of X_149_ was checked, and changes in the Tal protein were not sufficient to fully explain the slightly expanded host range of X_149_.

### Protein modularity in Krappator phages

Another differentiating factor between X_27_ and X_149_ was the presence of three deduced multi-domain proteins with similar predicted functions (TerS, Lys, and Replisome organizer) yet high modularity, suggesting the shuffling of protein modules (Fig. 3). The similarity was most pronounced in the C-terminal parts of the proteins, whereas the N-termini had diverged considerably between both Krappator phage genomes. We first observed divergence among the TerS proteins (Fig. S7). Owing to literature, the N-terminus and central sections of TerS are suggested to be responsible for binding properties to DNA and oligomerization mechanisms, respectively. We observed that TerS_X149_ was shorter than TerS_X27_ and shared identity to TerS from phages of LAB, including the Lactobacillus phage Phig1e (51). Yet, X_27_ and X_149_ phages had the same combinations of TerS C-terminal section and TerL protein types, suggesting their importance for the interaction between both subunits as proposed in other model phages (52). Whether and how the presence of divergent sequences impacts encapsidation remains to be established.

The second protein with apparent shuffling was the endolysin. Even though the EAD regions in Lys_X27_ (211 aa) and Lys_X149_ (216 aa) corresponded to GH25-like N-acetyl-β-D-muramidase domains, they belonged to distinct subfamilies (Conserved Domains Database [CDD] 06,417, GH25_lysA and CDD 06415, GH25_CpI1, respectively), supporting the idea that both endolysins are natural chimeras. Interestingly, we examined phenotypic differences in phage replication parameters and found that cell lysis upon infection of strain IOEBS277 by X_149_ was 24–48 h delayed compared to infection by X_27_ and X_28_.

Further evidence of modular shuffling was provided by the comparisons of the replisome organizers specified by X_27_ (ORF36) and X_149_ (ORF44). Both proteins shared a C-terminal DnaD domain with 45% identity to a hypothetical protein in the P335-type phage 6890 of *Lc. lactis* (locus tag D6890_040). Yet, the BBH of the N-terminal fragments of 148 aa corresponded to replication proteins associated with prophages in *Liq. satsumensis* (locus tag KBX31_11260) and *Weissella oryzae* (locus tag WOSG25_210040), respectively. The 5´AATGAC3´ sequence was repeated four times, resulting in the presence of a NDNDNDNDN possible linker between essential domains.

## DISCUSSION

While the biodiversity of LAB and their phages that thrive in dairy fermentations has been extensively explored over the last years (53), limited research has been conducted in plant-based fermented fruit and vegetable environments. The present work contributes to expanding the current knowledge about the biodiversity of phages infecting *O. oeni* during winemaking, through the characterization of the strictly lytic phage Krappator X_27_. Like all oenophages characterized to date, X_27_ has a siphophage morphotype. Its particularity lies in its high burst size (149 PFU/cell), as compared to the values measured in other oenophages in the same medium: 16–20 for P58I, 25 for Φ1002, 45 for OE33PA, and 55 for S1.1S (5). Of note, X_27_ is also able to lyse its host in grape juice. Furthermore, analysis of the samples from which the phage and its two counterparts X_28_ and X_149_ were isolated reveals that these lytic phages are present at every stage in the transformation of grapes into wine (must, AF, and MLF). Based on these observations, we carefully raise the possibility that Krappator phages may influence the overall microbial community composition and have adverse impacts on MLF kinetics and product quality.

Intriguingly, the three characterized Krappator phages were isolated from wines fermented from Merlot grapes, one of the main red grape varieties used in Bordeaux, which is also common in wine production in other parts of the world. Yet, no clear link between phage phylogeny and sample source, in terms of grape variety, was observed in previous studies. For example, Vinitor 162 and Vinitor 27 were obtained from two distinct varieties (9). The potential impact of the grape variety also lies in the fact that varieties are differentiated by the white or red character of the grapes. Solid evidence confirms that grape variety affects the concentration of total polyphenolic compounds (PCs) and their chemical diversity. Other important factors include grape maturity, environmental and agro-ecological conditions in vineyards, and winemaking technology. In our study, the characterization of X_27_ showed that red grape juice was more permissive to phage infection than white grape juice, regardless of the pH of the media. The concentration of total PCs is on average higher in the red varieties. Yet, interpreting our data remains a tricky task since (i) PCs have a complexity of effects in the cell and are notably responsible for dose-dependent alterations of microbial membranes, and (ii) musts/wines have a heterogeneous array of molecules, whose biological activities on cell growth and phage infectivity can be synergistic or antagonistic. Among wine PCs, research by Philippe et al. (54) has shown that some flavonoids prevent oenophage OE33PA from binding to its host and/or interfere with subsequent steps of the lytic cycle. In the first suggested hypothesis, flavonoids could interact with the phage adsorption device and occupy the receptor-binding sites, thereby blocking phage-host recognition. Alternately, the compounds may limit the synthesis and/or the access to CWPs (53, 54). A notable example is the impact of propolis on various LAB of the oral cavity. This bee product, enriched with a variety of flavonoids and phenolic acids, can inhibit the synthesis of the bacterial cell wall and reduce the expression and activity of various glycosyltransferases (55). Noteworthy, these enzymes play a pivotal role in assembling LAB CWPs of various composition and architecture, including cell wall teichoic acids, lipoteichoic acids, and CWPs which are embedded in the peptidoglycan layer (53). Rhamnose-containing CWPs have been associated with bacteriophage receptors for ovococcal LAB genera, including streptococci, enterococci, and lactococci (56). The *O. oeni* species has the potential to synthesize three types of homopolysaccharides (dextran, levan, β-glucan) as well as two heteropolysaccharides (57). Future work should now aim to determine the composition of the polymers, isolate bacterial insensitive mutants of *O. oeni*, and experimentally test the function of the potential host-binding domains identified in our AF2 structural model of the X_27_ host adhesion device. Like for many other LAB phages (10, 34, 58), this protein assembly contains a combination of CBMs likely functioning as receptor-binding domains. Moreover, the X_27_ Tal β-prisms could also be receptor-binding sites at their grooves that expose aromatic residues usually involved in interactions with polysaccharides. Noteworthy, in the L-shaped fibers of the siphophage T5, the cleavage of the intramolecular chaperone, which contains long β-hairpins interacting with upstream β-prims, exposes potential receptor-binding sites for the recognition of oligo-mannose units of the *E. coli* Lipopolysaccharides (LPS) (44). Although we could identify potential intramolecular chaperones’ catalytic dyads in X_27_, it remains to be determined whether self-cleavage occurs in viral particles, and, if so, what the structural trigger is. Since X_27_ harbors two consecutive β-prism-intramolecular chaperone motifs, both intramolecular chaperones, or at least the one of the first motif, could remain in infectious virions, thereby adding two additional potential receptor-binding sites to the CBM combination. The presence of β-prism-intramolecular chaperone motifs in the long Tals of the oenophages X_27_ and Vinitor 162 further highlights the modularity and structural diversity of LAB phages’ host adhesion devices (10, 34, 58, 59) evolved to ensure success in host detection in their ecological niches.

To date, very few active oenophages have been thoroughly characterized. Their analyses identified clear taxonomic boundaries and split them into two separate lineages of *cos*- and *pac*-type phages, named clusters 1 and 2, respectively (6, 11). Yet, key findings from our study reveal substantial novelty and lead us to propose the existence of a potentially novel genus of oenophages called “Krappavirus” within subcluster II. At the protein level, the similarity between virulent and temperate members of the proposed « Krappavirus *»* and *Sozzivirus* genera, respectively, extends essentially over the entire morphogenesis module of the phage genomes and may trace ancient gene flux (26). In contrast, Vinitor phages (subcluster II.1) are more distantly related and may be classified apart under a distinct and higher taxonomic rank (5, 6, 9), such as the temperate oenophages in cluster I.

Understanding the evolutionary history of Krappator phages is complicated, and ancient exchanges may have occurred between phages during co-infections, between prophages in the genome, or between prophages and infecting temperate phages. It is necessary to continue the work in order to determine whether the phage-encoded recombinases conserved among Krappator phages are involved in recombination events at specific sites between genomes (60). An interesting finding is that Krappator phages evolve by shuffling interchangeable functional modules in specific proteins. In agreement with recent data (61, 62), the domain-based protein modularity was observed in the endolysin, the replisome organizer, and the small terminase unit. Shuffling, therefore, appears to be an evolutionary strategy among Krappator phages to allow rapid adaptation to available hosts in their environment and to ensure their continued success.

## MATERIALS AND METHODS

### Bacterial strains and growth conditions

We used 20 strains of *O. oeni* originating from different beverages (wine, cider, kombucha, and water kefir). The *O. sicerae* strain was isolated from cider. We also included two strains of *O. kitaharae* previously obtained from Shochu, a traditional Japanese liquor (63), and from water kefir (64). Both *O. alcoholitolerans* strains (JP736 and 72/2) were provided by Prof. De Morais (Federal University Pernambuco, Brazil). All bacterial strains were obtained from the Centre de Ressources Biologiques Oenologiques (CRB Oeno, ISVV, Villenave d’Ornon, France).

Strains were routinely grown in MRS medium (Difco) at 25°C. Alternately, grape juice-containing media were used. Red Grape Juice medium contains 25% (vol/vol) of commercial red grape juice (Reflets de France, Carrefour, Bordeaux, France), 0.5% (wt/vol) of yeast extract, and 0.1% (vol/vol) of Tween 80. Commercial red grape juice was replaced with commercial white grape juice to prepare White Grape Juice medium (9). MLO medium (65) contains casein peptone (10 g/L), yeast extract (5 g/L), Tween 80 (1 g/L), sodium citrate (3.5 g/L), MgSO_4_ 7 H_2_0 (0.2 g/L), MnSO_4_ 7 H_2_0 (0.05 g/L), glucose (10 g/L), fructose (5 g/L), and tomato juice (100 mL). The pH of the media was adjusted to 4.8 using a concentrated HCl solution (37%). Solid media were prepared by adding 2.5% (wt/vol) of agar (Thermo Fisher Scientific, Bordeaux, France). All media were sterilized by autoclaving at 121°C for 15 min.

### Propagation of oenophages

Amplification of X_27_ on the bacterial host was carried out in MRS broth supplemented with MgSO_4_ (3.75 g/L) and CaCl_2_ (2.375 g/L) (MRS_Φ_) as follows. A liquid culture (10 mL at OD 0.3) was infected with 100 µL of phage lysate and incubated for 3 days at 25°C. It was centrifuged upon lysis at 10,000 *g* for 10 min. The supernatant was filtered through a 0.22 µm polyethersulfone membrane filter (4). Phages X_27_ and X_28_ were obtained from a collection of uncharacterized phages collected during previous surveys (8, 9) and stored inside infected cells at −80°C.

Tenfold serial dilutions of the fresh lysates were prepared in phage buffer (50 mM Tris-HCl pH 7.5; 0.1 M NaCl; 8 mM MgSO_4_). Phages were enumerated using the classical double-layer plating technique (4). MRS_Φ_ agar plates were incubated in jars under anaerobic conditions using GasPak EZ sachets (Thermo Fisher Scientific, Bordeaux, France) for 5–7 days at 25°C.

### Optimal multiplicity of infection in liquid infection of IOEBS277 by X_27_

The IOEBS277 strain was grown until an OD of 0.05 was reached in MRS_Φ_ broth (100 mL). The culture was distributed into 9 mL aliquots. Volumes of 1 mL of phage X_27_ and dilutions thereof were added to the cultures to yield MOIs ranging from 10^−2^ to 10^−6^. A control with no added phage was prepared. All tubes were incubated at 25°C and visually examined after 3 days (9).

### TEM observations

A 50 mL phage lysate was centrifuged at 20,000 *g* for 2 h. The pellet was air-dried and resuspended in 50 µL of SM buffer (100 mM NaCl, 25 mM Tris-HCl pH 7.5, 8 mM MgSO_4_) (66). A volume of 10 µL (~10^9^ PFU/mL) was deposited on a carbon-coated copper grid for 30 s, then colored with uranyl acetate (saturated in water pH 4.5) for 30 s. The phage was visualized using a Hitachi H7650 electron microscope operated at 80 kV.

### Host spectrum of X_27_

The X_27_ phage lysate was dialyzed against 500 mL of SM buffer for 3 h at 4°C, with one buffer change. A Float-A-Lyzer G2 Dialysis Device Molecular weight cutoff (MWCO) 100 kDa (Sigma-Aldrich, France) was used according to manufacturer’s directions. The propagation of the phage was tested on a panel of *O. oeni* and *O. kitaharae* strains as follows. The phage lysate was spotted (8 µL) in duplicates at eight concentrations (10^9^ to 10^2^ PFU/mL) onto those selected potential hosts. The resistance level of bacterial strains to the phage was expressed using the efficiency of plating (EOP) ratio. The EOP was defined as the ratio between PFUs/mL obtained on each putative resistant strain and PFUs/mL obtained on the strain initially used for the phage propagation (*O. oeni* IOEB S277). The EOP value was classified as “high production” when the ratio ranged from 1 to 10^−1^. An EOP between 10^−2^ and 10^−7^ was considered to be of “medium production” efficiency. Finally, an EOP under 10^−8^ was classified as inefficient, and corresponding strains were considered as resistant strains (54, 66).

### Adsorption experiments

Cells in early exponential phase growth (2 mL with an OD_600_ of 0.2) were centrifuged at 10,000 *g* for 10 min. The pellet containing approximately 8 × 10^8^ Colony Forming Unit (CFUs) was resuspended in 1,600 μL of fresh MRS_Φ_ broth. The phage lysate was placed at room temperature for 15 min. A volume of 400 µL of diluted lysate (corresponding to ~2.7 × 10^6^ PFUs) was prepared and added to the 1,600 µL of sample, to yield an MOI of 0.003. The 2 mL sample was immediately homogenized, and a volume of 200 µL (T0) was immediately collected, centrifuged (10,000 *g*, 5 min), and filtered. The remainder of the sample was equally distributed in four tubes which were placed at 25°C in a water bath. They were periodically removed after 20, 30, 40, and 60 min and centrifuged. All supernatants were serially diluted and titrated on strain IOEBS277 to give the concentrations of free (unbound) phages over time (54).

### One-step growth and burst size

A 2 mL adsorption assay was prepared as described above. A volume of 200 µL was immediately removed at T0, centrifuged, and filtered. The infected culture was incubated at 25°C for 60 min to allow phage adsorption. After this period, removal of unadsorbed virions was performed by centrifugation of the 1.8 mL sample (10,000 *g* for 5 min at 4°C). Supernatants collected at T0 and T60 were titrated in order to measure adsorption rate. The pellet containing infected cells was gently resuspended in 10 mL of pre-heated MRS_Φ_ broth. A volume of 500 µL was removed and added to 29.5 mL of MRS_Φ_ broth. The diluted infected cells were distributed in 2 mL samples, which were incubated at 25°C. Samples were withdrawn over time for their application in an infectious center assay following centrifugation.

The burst size is the ratio of phages produced compared to the initial number of infective centers. It was calculated as the ratio of the final count of liberated virions at the end of the burst period to the initial count of infected bacterial cells at the beginning of the latent period. The lysis time was the number of hours before the increase in plaque number occurred. Three replicate experiments were conducted to estimate adsorption rate, burst size, and lysis time (66).

### Genome sequencing, genomic comparisons, and phylogenetic analyses

The phage lysate was concentrated by ultracentrifugation, and double-stranded DNA was extracted as described previously (4, 5). Whole-genome sequencing was performed at the Genome-Transcriptome facility of Bordeaux (https://pgtb.cgfb.u-bordeaux.fr). DNA libraries were prepared using the Nextera XT DNA library preparation kit (Illumina, San Diego, CA). Genomic DNA was sequenced by Illumina MiSeq using 2 × 250 bp paired-end libraries. Reads were assembled using SPAdes (version 3.14.1) (67) with default parameters (read correction and assembler). The assembly of the whole-genome sequences was verified using *Hin*dIII, *Eco*RI, and *Bam*HI restriction profiles of phage DNAs and gel electrophoresis.

The sequences of the assembled phages were then used to predict ORFs with RAST (http://rast.nmpdr.org/) and Pharokka (https://github.com/gbouras13/pharokka) (68). The latter includes the tRNAscan-SE program (http://lowelab.ucsc.edu/tRNAscan-SE/) (69).

The *in silico*-translated protein sequences were used as queries to search for sequence homologs in the non-redundant protein database at the National Centre for Biotechnology. Deduced proteins were examined for their function using BLAST v.2.10.0 and a cutoff E value of 0.001. Searches for distant homologs were performed using HHpred (70) against different protein databases, including PFAM (Database of Protein Families), PDB, CDD, PHROG, and Clusters of Orthologous Groups, which are accessible via the HHpred website. Searches against the CDD database at NCBI were also performed using CD-search (71).

The online analysis tools Arnold (72), which predict the existence and location of Rho-independent transcription terminators employing RNAmotif and ERPIN complementary programs and TMHMM (73), were used. Average nucleotide identity was obtained using VIRIDIC (version 1) to determine the overall similarity between two genomic sequences and then visualized by pheatmap in R (48, 49). For genomes, all pairwise comparisons of the nucleotide sequences were conducted using the Genome-BLAST Distance Phylogeny method using VICTOR under settings recommended for prokaryotic viruses as earlier published (9). Genome homology was visualized using Clinker (14).

### PCR screening of Krappavirus phages in our collection

Phage lysates were typed by PCR as reported before (9, 11) using specific primers TMP7-F 5´-tagaaacatatacggcacagcc-3´ and TMP7-R 5´-tctagaaccttgttcaaagcgt_3´), which were designed to amplify a portion of the tape measure protein of Krappavirus phages, without any cross-reactivity with other oenophages.

### Host adhesion device structure prediction

X_27_ protein sequences encoded by genes located in between the *tmp* and *lysin-holin* were submitted to HHpred (74). Structure predictions were performed for the Dit and Tal proteins using an AlphaFold2 Github notebook (https://colab.research.google.com/github/deepmind/alphafold/blob/main/notebooks/AlphaFold.ipynb#scrollTo=XUo6foMQxwS2) (32), enabling structure predictions of multimers. The structure of Dit was predicted as a monomer and as a hexamer. Due to memory limitations, the long Tal was split into distinct stretches with large overlapping regions for later assembly. Monomeric and trimeric structures were predicted for each of them. Structure of the full-length Tal was obtained by superimposing the overlapping regions onto each other using Coot (75). AF2 provides an index called predicted local distance difference test (pLDDT) estimating the prediction accuracy along the protein chain, from 0 to 100 (best). In practice, pLDDT values over 80–90 compare to average-resolution crystal structures. The pLDDT values, which are stored in the PDB files as B-factors, were plotted using Excel. The final predicted domain structures were submitted into the Dali server (76) to identify the closest structural homologs in the PDB. Sequence alignments were performed with Multalin (77) and ESPript (78). Structure analyses and visual representations were performed with ChimeraX (79).

## Data Availability

The full genomes of *O. oeni* phages X_27_and X_149_ were deposited in GenBank under the accession numbers PQ156465 and PQ156466. Structural models are deposited in the Zenodo repository under the identifiers AA, BB.

## SUPPLEMENTAL MATERIAL

Supplemental material spectrum.02588-24-s0001.docx Figures S1 to S7; Tables S1 and S2.

ASM does not own the copyrights to Supplemental Material that may be linked to, or accessed through, an article. The authors have granted ASM a non-exclusive, world-wide license to publish the Supplemental Material files. Please contact the corresponding author directly for reuse.
