# Supplementary material for: Characterization of strictly lytic phages infecting Oenococcus oeni from Merlot wines and proposal of a new genus
Source: Microbiol Spectr. 2025 Aug 12;13(9):e02588-24. doi: 10.1128/spectrum.02588-24 (PMC12403621; doi:10.1128/spectrum.02588-24)
Supplement: Supplemental material — Figures S1 to S7; Tables S1 and S2. [file spectrum.02588-24-s0001.docx]

**
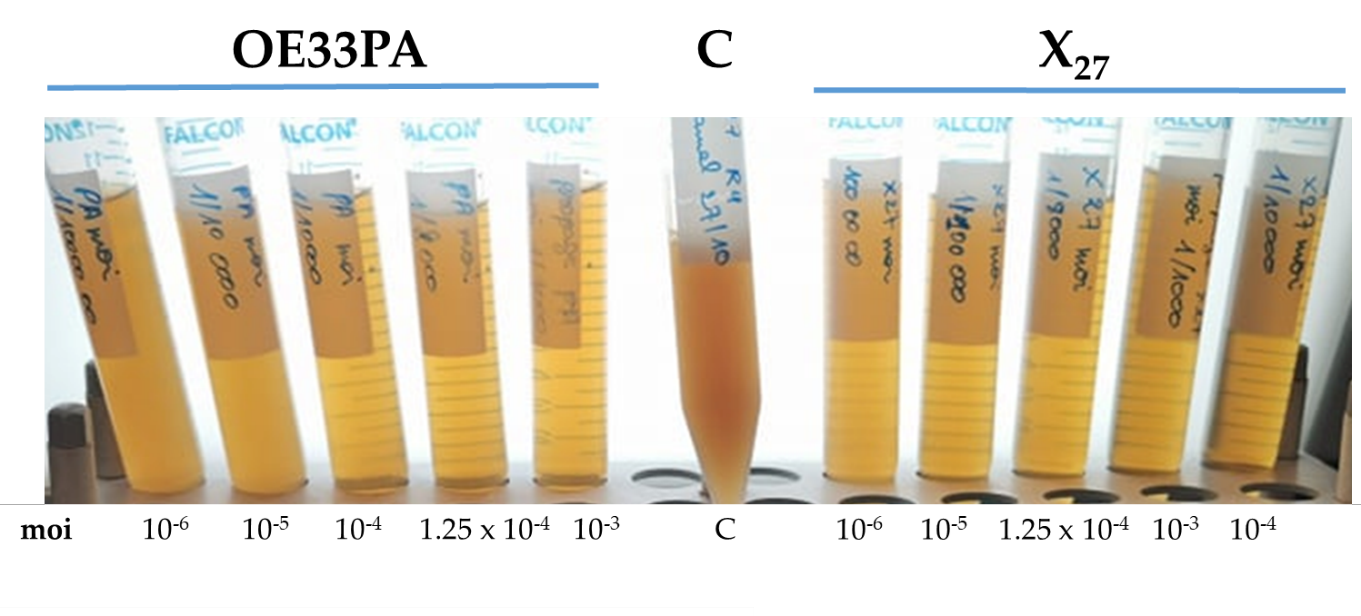
**

**Fig. S1.** Cultures of *O. oeni* IOEB277 in MRS broth without bacteriophages (C, for control) and with bacteriophages OE33PA (left) and Krappator X_27_ (right) at different MOI after 72 h incubation at 25°C.


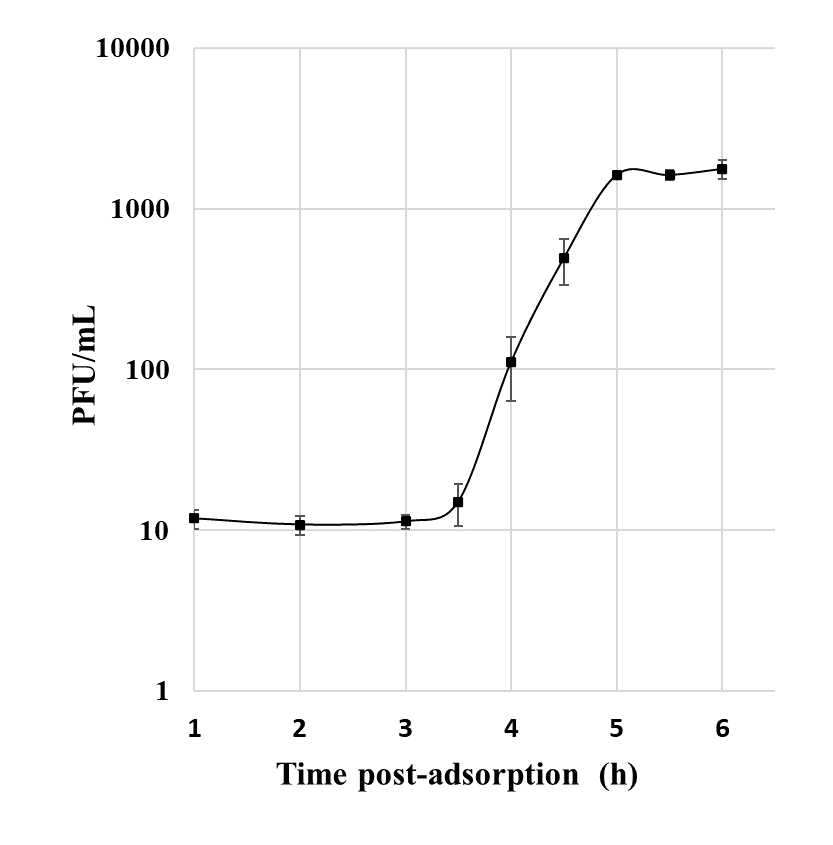


**Fig. S2** One-step growth curve experiments of Krappator X_27_ in MRS broth. The results show the mean of 3 experiments. Bars: standard deviation.

**
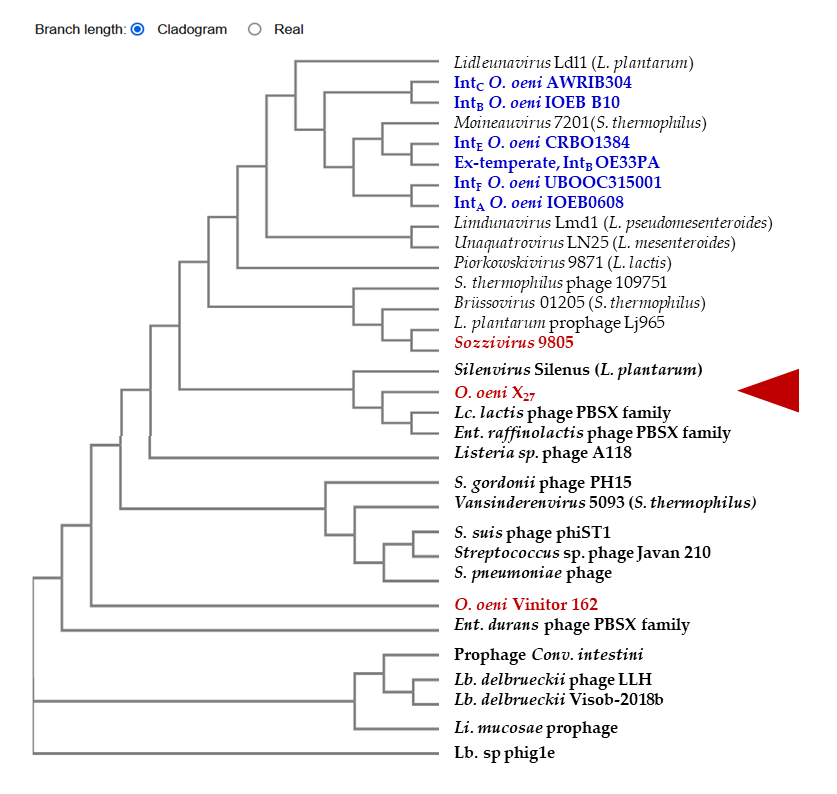
**

**Fig. S3** ClustalW alignments of the TerL protein sequences of phages infecting lactic acid bacteria. Clusters I and II of oenophages are represented in blue and red, respectively. References are according to previous work (9).

**
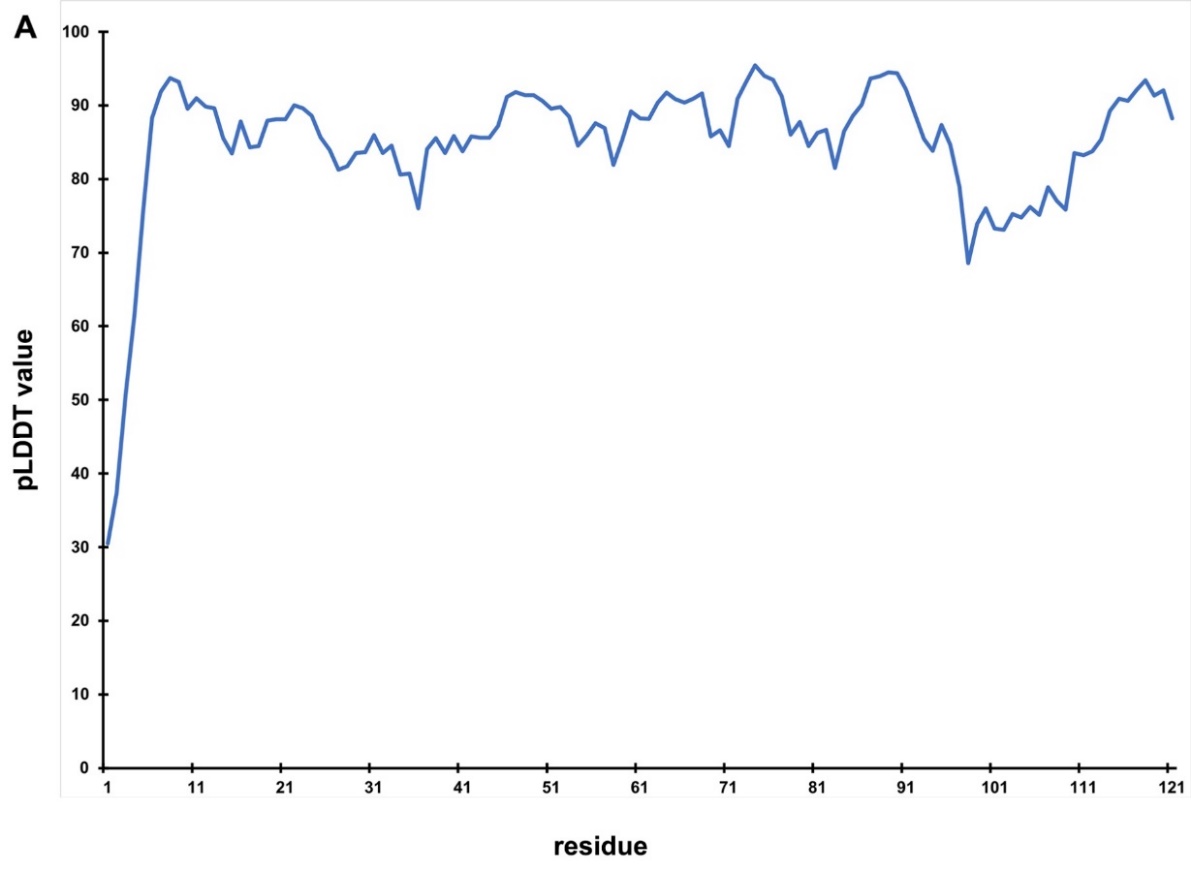
**

**
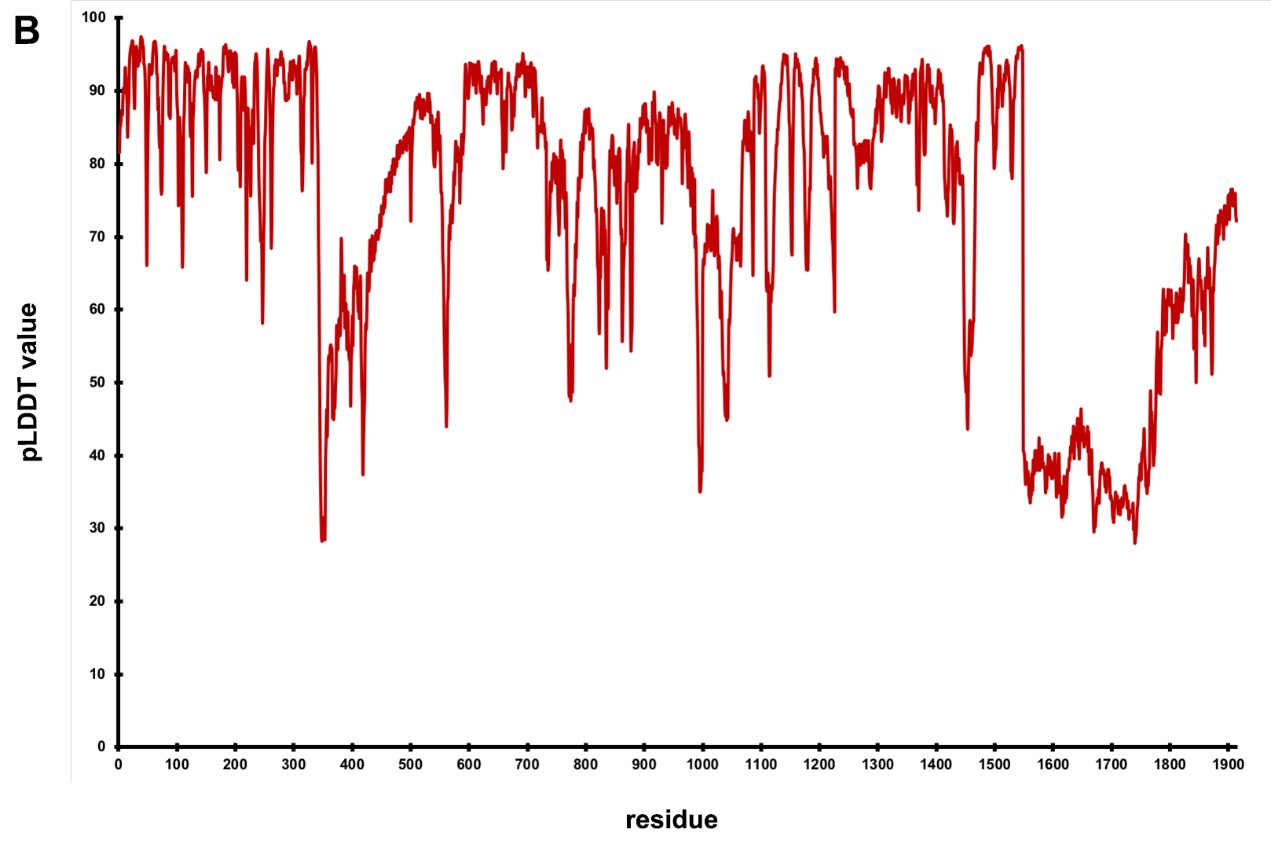
**

**Fig. S4** Plots of pLDDT (predicted local distance difference test) values for structure predictions. A) pLDDT values for each residue along the X_27_ Dit sequence are plotted. B) pLDDT values for each residue along the X_27_ Tal sequence are plotted.


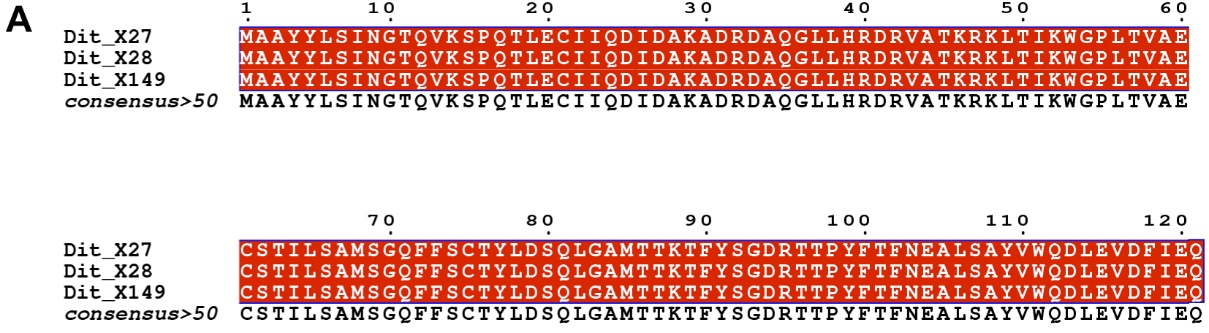


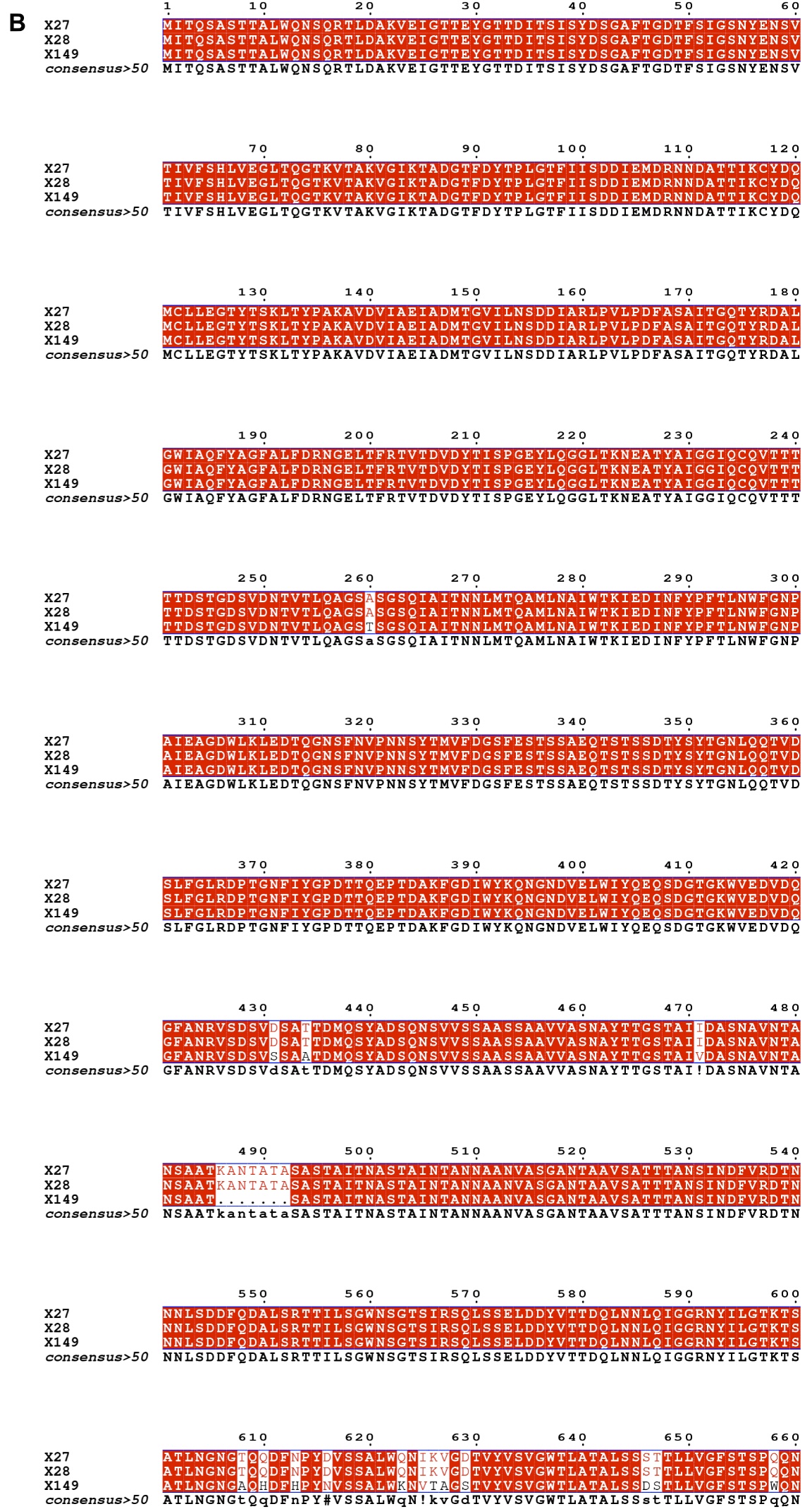


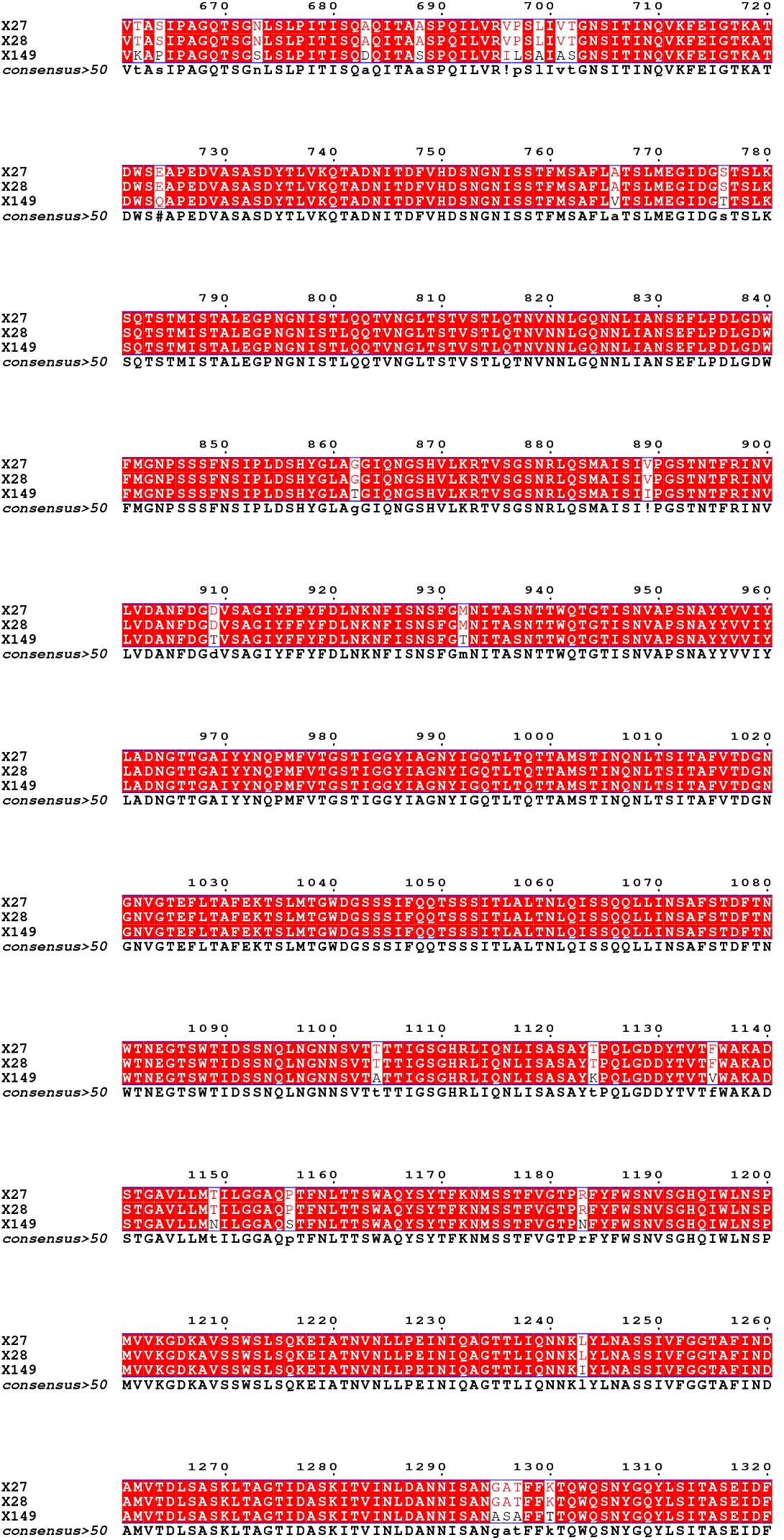


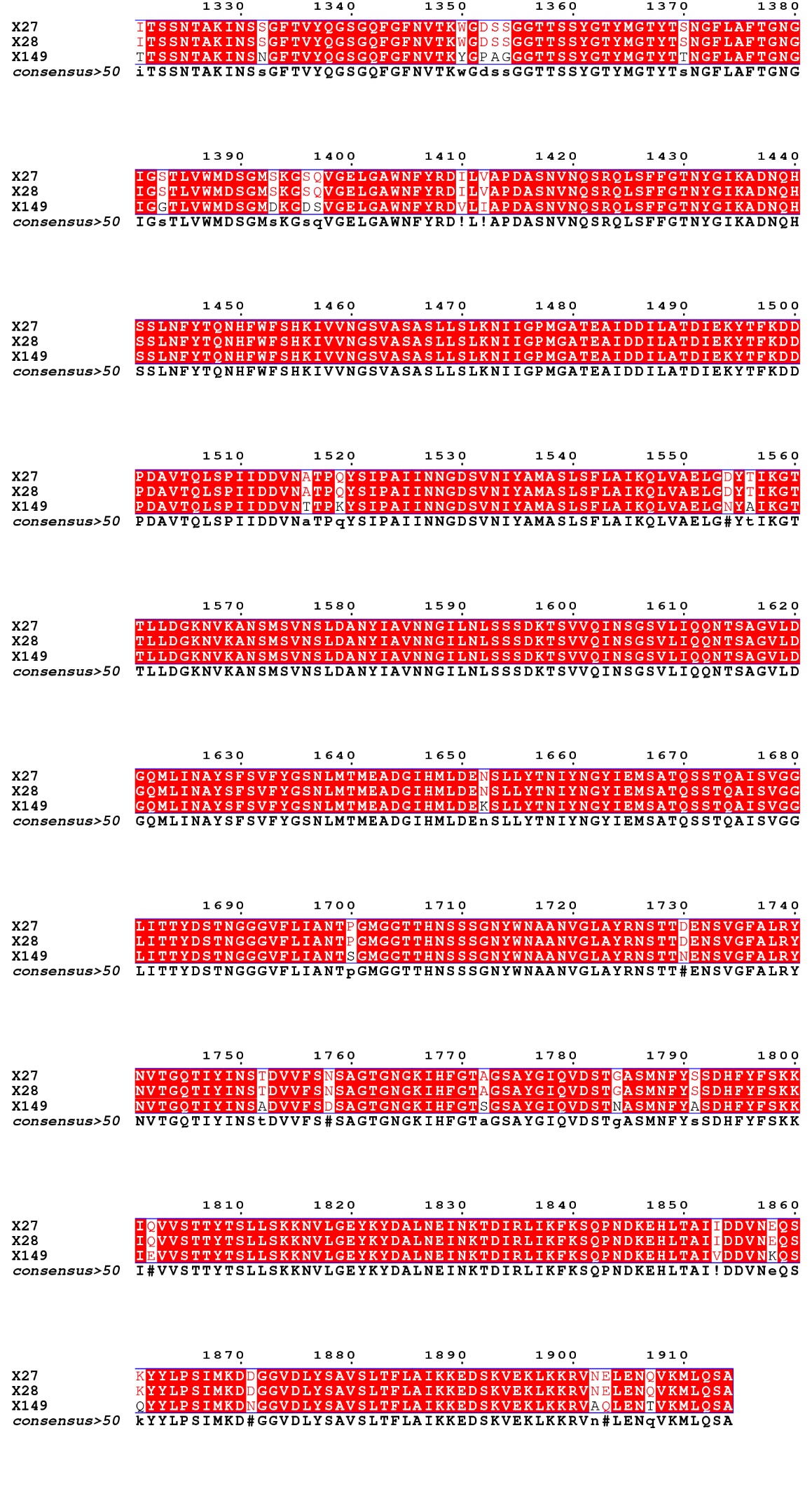


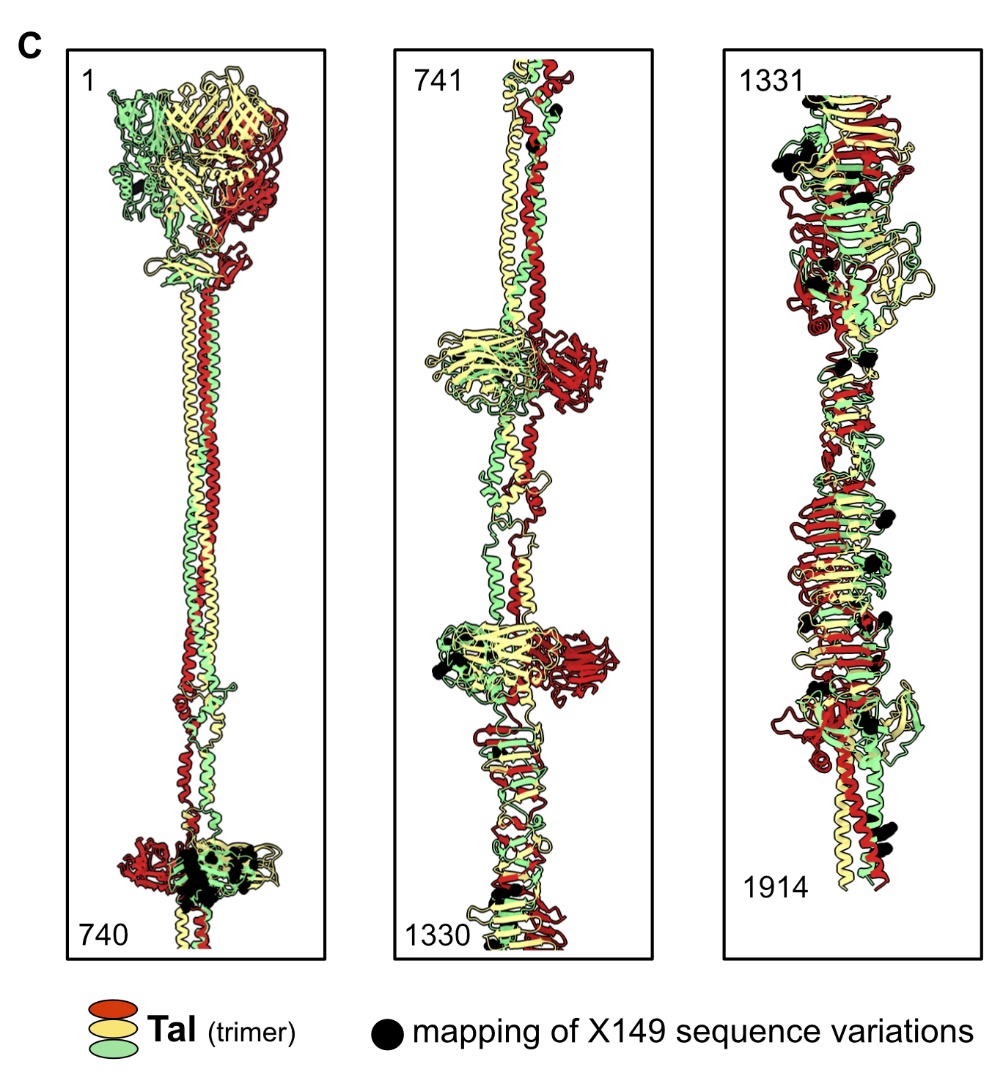


# **Fig. S5** Amino acid alignment of Dit and Tal proteins from Krappator phages and mapping of X_149_ sequence variations. A) Multiple alignment of X_27_, X_28_ and X_149_ Dit sequences. B) Multiple alignment of X_27_, X_28_ and X_149_ Tal sequences. C) Mapping of X_149_ Tal sequence variations on the X_27_ Tal predicted structure.

**
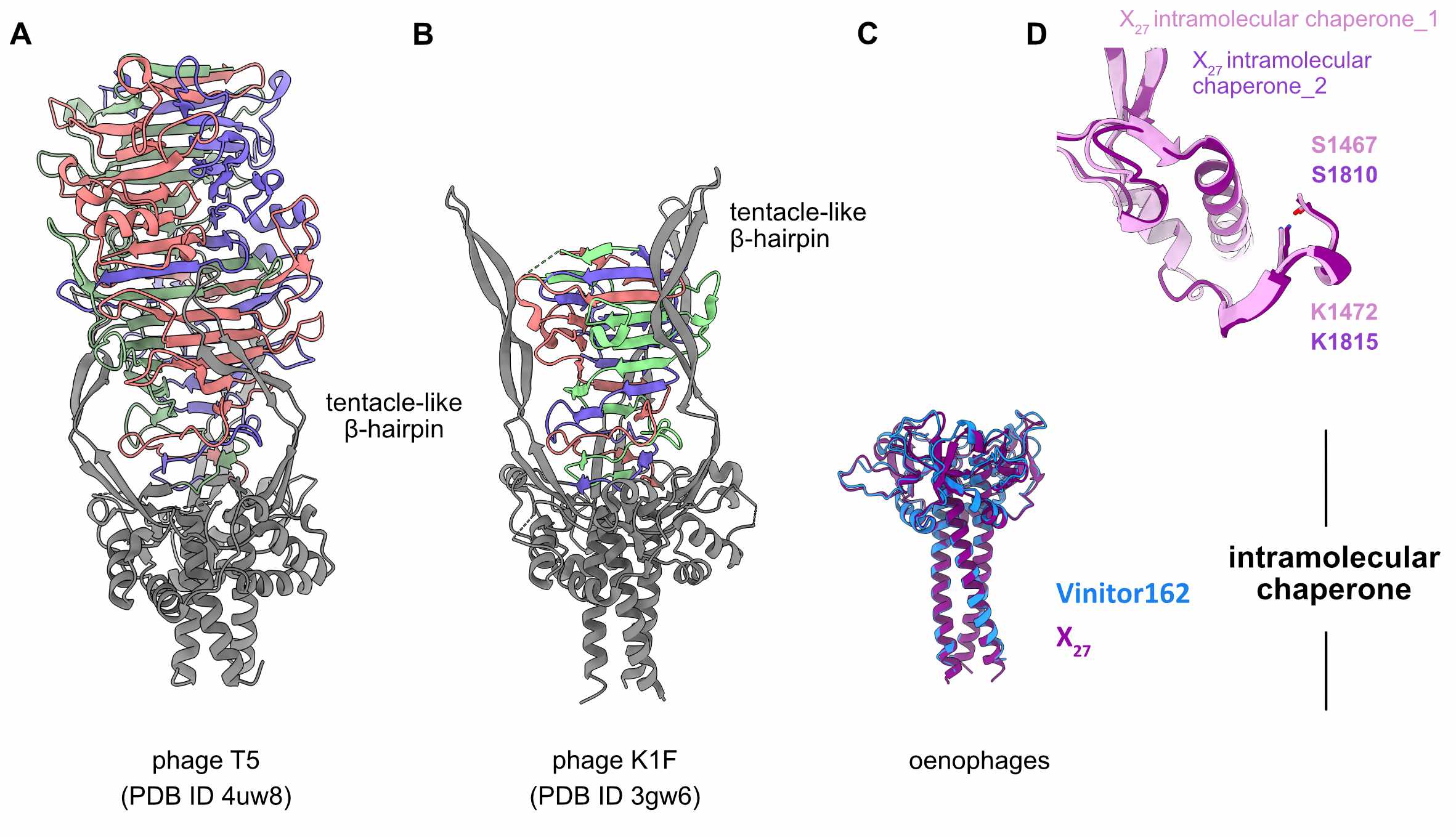
**

**Fig. S6 Structural analyses of X_27_’s intramolecular chaperones.** A) Ribbon representation of the crystal structure of the phage T5 intramolecular chaperone (grey) with its tentacles contacting the upstream β-prism (green, purple, pink) in its L-shaped tail fiber. B) Ribbon representation of the crystal structure of the phage K1F intramolecular chaperone (grey) with its tentacles contacting the upstream β-prism (green, purple, pink) in its tail spike protein. C) Superposition of the X_27_ and Vinitor_162_ intramolecular chaperone predicted structures. D) Potential catalytic dyads of the X_27_ intramolecular chaperones are shown as sticks.


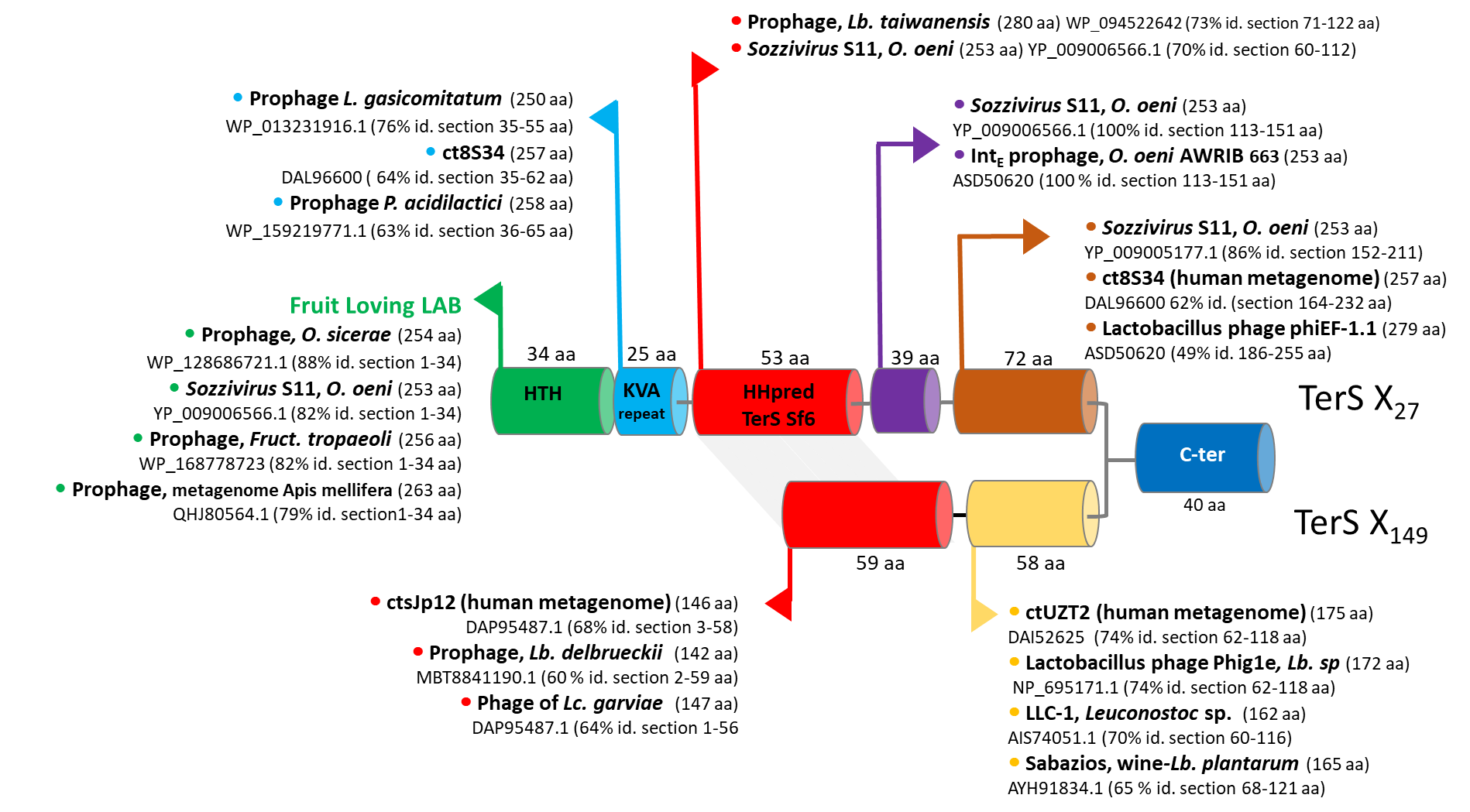


**Fig. S7** Long (TerS_X27_) and short (TerS_X149_) small terminase subunits of Krappator phages and comparison with other TerS with from various phages infecting lactic acid bacteria. HTH, Helix-Turn-Helix.

**Supplemental Table S1**

| **Gene** | | | | | **Phrog** | | **Blast/HHpred** | | **Predicted function** | **Module** |
| --- | --- | --- | --- | --- | --- | --- | --- | --- | --- | --- |
|  | **start** | **stop** | **size** | **F** | **nber** | **e-value** | **features** | **e-value** |  |  |
| 01 | 49 | 843 | 795 | + | 2606 | 2,98E-46 |  |  | terminase small subunit | head and packaging |
| 02 | 827 | 2137 | 1311 | + | 3717 | 2,61E-69 |  |  | terminase large subunit |  |
| 03 | 2156 | 3661 | 1506 | + | 64 | 5,29E-80 |  |  | portal protein |  |
| 04 | 3621 | 3914 | 294 | + | 4478 | 3,76E-20 |  |  | peptidase |  |
| 05 | 3916 | 5082 | 1167 | + | 115 | 2,43E-54 |  |  | minor head protein |  |
| 06 | 5108 | 5290 | 184 | + | none | none |  |  | HP | unknown function |
| 07 | 5446 | 5613 | 168 | + | none | none |  |  | HP |  |
| 08 | 5648 | 5776 | 129 | + | none | none |  |  | HP |  |
| 09 | 5760 | 6458 | 699 | + | 172 | 7,18E-18 |  |  | head scaffolding protein | head and packaging |
| 10 | 6476 | 6847 | 372 | + | 909 | 9,41E-16 |  |  | major head protein |  |
| 11 | 6866 | 7924 | 1059 | + | 29 | 1,07E-31 |  |  | major head protein |  |
| 12 | 7944 | 8249 | 306 | + | 132 | 2,77E-06 |  |  | head-tail adaptor | connector |
| 13 | 8246 | 8605 | 360 | + | 2168 | 3,64E-47 | *L. ghanensis* KRM06969.1 | 2,00E-39 | head closure knob protein |  |
| 14 | 8598 | 9149 | 552 | + | 5 | 1,53E-06 |  |  | tail completion or neck1 protein |  |
| 15 | 9149 | 9517 | 363 | + | none | none | *L. johnsonii* NP_958591.1 | 2,00E-39 | tail completion protein | tail |
| 16 | 9523 | 9978 | 456 | + | 158 | 3,14E-20 |  |  | major tail protein |  |
| 17 | 10063 | 10461 | 399 | + | 183 | 1,87E-15 |  |  | tail chaperone protein |  |
| 18 | 10500 | 10841 | 342 | + | 2066 | 9,07E-43 |  |  | HP | unknown function |
| 19 | 10874 | 15730 | 4857 | + | 1535 | 3,25E-152 |  |  | tail length tape measure protein | tail |
| 20 | 15746 | 16111 | 366 | + | 1194 | 7,43E-24 | *L. ghanensis* WP_057871071.1 | 3,00E-63 | Dit |  |
| 21 | 16129 | 21873 | 5745 | + | 22212 | 8,71E-188 | phage B10  *O. oeni* KGH57015.1 | 0,00E+00 | Tal |  |
| 22 | 21915 | 22328 | 414 | + | 13237 | 3,62E-58 |  |  | HP | unknown function |
| 23 | 22329 | 22577 | 249 | + | 2566 | 5,28E-08 |  |  | HP |  |
| 24 | 22705 | 23103 | 399 | + | none | none |  |  | HP |  |
| 25 | 23100 | 23504 | 405 | + | none | none |  |  | HP |  |
| 26 | 23573 | 23836 | 264 | + | none | none |  |  | HP |  |
| 27 | 23912 | 24247 | 336 | + | none | none |  |  | HP |  |
| 28 | 24263 | 25354 | 1092 | + | 291 | 5,12E-49 |  |  | endolysin | lysis |
| 29 | 25419 | 25763 | 345 | + | 5702 | 8,21E-15 |  |  | Holliday junction resolvase | DNA, RNA and nucleotide metabolism |
| 30 | 25940 | 26821 | 882 | + | 148 | 3,00E-42 |  |  | RecT-like ssDNA annealing protein |  |
| 31 | 26772 | 27668 | 897 | + | 1227 | 1,07E-52 |  |  | exonuclease |  |
| 32 | 27678 | 27944 | 267 | + | none | none |  |  | HP | unknown function |
| 33 | 27941 | 28369 | 429 | + | 44 | 3,85E-36 |  |  | single strand DNA binding protein | DNA, RNA and nucleotide metabolism |
| 34 | 28454 | 28714 | 261 | + | 3 | 5,50E-09 |  |  | transcriptional repressor |  |
| 35 | 28674 | 29432 | 759 | + | 16986 | 5,84E-66 |  |  | HNH endonuclease |  |
| 36 | 29419 | 30318 | 900 | + | 1701 | 6,17E-39 |  |  | Replisome organizer |  |
| 37 | 30311 | 30814 | 504 | + | none | none |  |  | HP | unknown function |
| 38 | 30830 | 31183 | 354 | + | none | none |  |  | HP |  |
| 39 | 31173 | 31388 | 216 | + | none | none |  |  | HP |  |
| 40 | 31351 | 31590 | 240 | + | none | none |  |  | HP |  |
| 41 | 31599 | 32312 | 714 | + | 398 | 2,83E-47 | prophage ct8S34 *L.mesenteroides* DAL96604.1 | 4,00E-30 | HNH endonuclease | DNA, RNA and nucleotide metabolism |
| 42 | 32341 | 32430 | 90 | + | none | none |  |  | HP | unknown  function |
| 43 | 32414 | 33055 | 642 | + | none | none |  |  | HP |  |
| 44 | 33114 | 33674 | 561 | + | 5105 | 1,49E-25 |  |  | HP |  |
| 45 | 33674 | 33979 | 306 | + | none | none |  |  | HP |  |
| 46 | 33963 | 34373 | 411 | + | 98 | 4,36E-14 |  |  | HP |  |
| 47 | 34330 | 34881 | 552 | + | 15471 | 1,00E-08 | Satyr phage YP_009797688.1 | 3,00E-84 | NinB-like HNH endonuclease | DNA, RNA and nucleotide metabolism |
| 48 | 34881 | 35147 | 267 | + | 986 | 6,10E-22 |  |  | RNA-binding protein |  |
| 49 | 35157 | 35378 | 222 | + | none | none |  |  | HP | unknown function |
| 50 | 35398 | 35664 | 267 | + | none | none |  |  | HP | unknown function |
| 51 | 35664 | 35804 | 141 | + | none | none |  |  | HP | unknown function |
| 52 | 35801 | 36172 | 372 | + | none | none |  |  | HP | unknown function |
| 53 | 36169 | 36330 | 162 | + | none | none |  |  | HP | unknown function |
| 54 | 36305 | 36460 | 156 | + | none | none |  |  | HP | unknown function |
| 55 | 36427 | 36801 | 375 | + | 1492 | 1,69E-20 |  |  | HNH endonuclease | DNA, RNA and nucleotide metabolism |
| 56 | 36803 | 36892 | 90 | + | none | none |  |  | HP | unknown function |
| 57 | 36920 | 37159 | 240 | + | none | none |  |  | HP |  |
| 58 | 37159 | 37332 | 174 | + | none | none |  |  | HP |  |
| 59 | 37342 | 38202 | 861 | + | none | none |  |  | HP |  |
| 60 | 38259 | 38552 | 294 | + | none | none |  |  | HP |  |
| 61 | 38549 | 38704 | 156 | + | 37779 | 5,78E-30 |  |  | HP |  |
| 62 | 38707 | 39051 | 345 | + | 98 | 3,25E-06 | phage ctb77 DAH24246.1 | 9,00E-03 | YopX-like protein |  |
| 63 | 39103 | 39420 | 318 | + | none | none |  |  | HP |  |
| 64 | 39427 | 39525 | 99 | + | none | none |  |  | HP |  |
| 65 | 39552 | 39644 | 93 | + | none | none |  |  | HP |  |
| 66 | 39670 | 39999 | 330 | + | none | none |  |  | HP |  |
| 67 | 40089 | 40229 | 141 | + | none | none |  |  | HP |  |
| 68 | 40292 | 40576 | 285 | + | none | none |  |  | HP |  |
| 69 | 40573 | 40719 | 147 | + | none | none |  |  | HP |  |
| 70 | 40806 | 41330 | 525 | + | 248 | 8,58E-14 |  |  | ArpU-like transcriptional activator | transcription regulation |
| 71 | 41323 | 41463 | 141 | + | none | none |  |  | HP | unknown function |
| 72 | 41633 | 41544 | 90 | - | none | none |  |  | HP |  |

**Supplemental Table S2.** Structural homologs of the X_27_ Dit and Tal proteins and of their domains (top hits from the Dali sever).

| **Protein**  **(residues)** | **Dali server outputs** | | |
| --- | --- | --- | --- |
|  | **PDB ID** | **Z-score^#^** | **rmsd (Å)^$^** |
| **Dit**  (1-121) | 2wzp (siphophage p2)  8xcg (siphophage Lambda) | 8.3  8.2 | 2.9 (106/121)  2.4 (98/121) |
| **Tal** |  |  |  |
| **N-terminal domain**  (1-340) | 3cdd (prophage MuSO2)  2p5z (T6 Secretion System) | 18.8  16.8 | 3.5 (257/340)  3.3 (241/340) |
| **CBM_1**  (598-724)  **CBM_2**  (838-980)  **CBM_3**  (1080-1206) | 2zew  (CBM16, CAZy nomenclature) | 10  14.5  16 | 2.5 (109/147)  2.9 (137/155)  2 (127/137) |
| **β-helices-β-prism_1**  (1274-1465) | 3pqi (phage Phi92) | 7.2 | 10.6 (108-175) |
| **Intramolecular chaperone _1**  (1466-1533) | 7dc3  (eukaryotic transc. regulation factor)  4uw8 (phage T5) | 7.5  6.2 | 2.2 (97/105)  2.7 (92/105) |
| **β-helices-β-prism_2**  (1565-1809) | 6orj  (phage PhiKZ) | 9.1 | 5.5 (126/227) |
| **Intramolecular chaperone _2**  (1810-1914) | 7dc3  (eukaryotic transc. regulatory factor)  4uw8 (phage T5) | 8.6  5.9 | 1.9 (108/123)  2.7 (94/123) |

**#**: The z-score is a measure of structural similarity.

**$:** Root mean square deviation on Cα atoms (aligned atoms/total atoms).
